# The impact of national savings on economic development: a focused study on the ten poorest countries in Sub-Saharan Africa

**DOI:** 10.1038/s41598-024-67228-x

**Published:** 2024-07-15

**Authors:** Ndukwe Ibe

**Affiliations:** School of Business and Creative Industries, University of the West of Scotland, Paisley Campus, Paisley, Scotland UK

**Keywords:** National savings, Economic development, Sub-Saharan Africa, Human development index, Panel data analysis, Statistics, Software

## Abstract

The purpose of this study is to examine the impact of national savings on economic development, as measured by the Human Development Index (HDI), Inequality-adjusted HDI (iHDI), and Multidimensional Poverty Index (MPI), in ten of the poorest countries in Sub-Saharan Africa. The study employs a sequential Generalized Method of Moments (GMM) analysis to address potential endogeneity issues and account for the dynamic nature of the relationships, covering the period from 2009 to 2019. The findings reveal a complex relationship between national savings and the selected development indicators. While national savings exhibit positive impacts on HDI and iHDI, the results are not consistently statistically significant across all the sequential models. However, the analysis suggests that national savings have a positive influence on reducing multidimensional poverty, as measured by MPI, particularly when effectively channeled into productive investments. The study also highlights the significant positive impact of government expenditure and foreign direct investment (FDI) on human development, underscoring the importance of strategic public investments and foreign capital. The results suggest that while national savings are crucial, their effective utilization is essential for enhancing human development indices. Strategic investments in public goods and foreign capital are also important. The mixed effects of inflation and official development assistance (ODA) emphasize the need for stable economic policies and effective utilization of foreign aid. The modest positive impact of institutional quality suggests that improvements in governance and institutional frameworks can contribute to human development. The findings underscore the need for policies promoting financial inclusion, efficient public expenditure, foreign direct investment, and stable macroeconomic conditions to leverage national savings for economic development. The study’s findings provide valuable insights for policymakers in Sub-Saharan Africa, highlighting the need for comprehensive strategies that leverage national savings, public expenditure, and foreign investment to drive sustainable economic development and poverty reduction.

## Introduction

The economic landscape of Sub-Saharan Africa is marked by a blend of challenges and opportunities, where national savings play a crucial role in determining the region's developmental trajectory. National savings, encompassing the total savings within a country from both public and private sectors, are fundamental for funding investments essential for economic growth and development. Scholars have consistently found that low savings rates are closely associated with developmental and welfare challenges, while higher savings rates can boost capital accumulation and potentially foster sustained economic growth^1–3^. Beyond fiscal accumulation, the significance of national savings extends to investment, financial stability, access to credit, and the broader well-being of society. The essence of savings, which involves postponing current consumption for future capital formation, is vital for enhanced production^4^.

Various theoretical frameworks, such as Rostow’s Stages of Growth, the Harrod-Domar Growth Model, and the Solow Growth Model, provide different insights into the connection between economic development, savings, and investment. Rostow’s model outlines the stages of economic development in relation to savings and investment rates (Rostow 1950s–1960s). The Harrod–Domar model underscores the importance of savings for capital accumulation and its influence on growth, particularly in labor-surplus developing countries^5,6^. The Solow Model, on the other hand, discusses how savings rates, along with population growth and technological progress, impact output changes, highlighting the role of savings in long-term economic prosperity^7^. Nordhaus^8^ posited that failing to save for the future typically results in economic and living standards decline, suggesting that increased savings could elevate investment, thereby boosting output, real wages, and living standards. Building on these theoretical foundations, this research delves into the intricate dynamics between savings and economic development. It is well-documented that nations maintaining high savings rates often enjoy robust economic development over time. Economic development is about harnessing capacities that push societal progress by leveraging the potentials of individuals, firms, and communities, crucial for creating conditions favorable to growth and securing a nation’s economic destiny^9^. Particularly in developing regions like Sub-Saharan Africa, governments grapple with insufficient reserves and investments, leading to a scarcity of business development funds and persistent poverty, despite previous revenues^10,11^. This study focuses on the challenges faced by these developing nations, with a special emphasis on Sub-Saharan Africa, where a lack of emphasis on national savings has led to an increased reliance on foreign debts and a shortage of developmental project funds. Despite abundant natural resources, the region continues to lag economically due to inadequate savings and financial management^12–14^.

## Research objectives and hypothesis

The objectives of this study are to examine the impact of national savings on the Human Development Index (HDI), Inequality-adjusted Human Development Index (iHDI), and Multidimensional Poverty Index (MPI) across Sub-Saharan African, and to analyze the role of government expenditure, inflation, official development assistance, and foreign direct investment in shaping the relationship between national savings and economic development. Focusing on ten countries with the highest poverty rates in Sub-Saharan Africa, this research aims to provide empirical insights into the savings-development nexus. The choice of HDI, iHDI, and MPI as indicators allows for a multidimensional assessment of economic development, beyond mere income metrics. By shedding light on this relationship, the study seeks to contribute to the formulation of policies that leverage national savings for socio-economic improvement. The study hypothesizes that higher national savings rates impact economic development in Sub-Saharan Africa, as measured by the Human Development Index (HDI), Inequality-adjusted HDI (iHDI), and Multidimensional Poverty Index (MPI). Specifically, we expect that: H1: National savings rates impact HDI scores; H2: National savings rates impact iHDI scores.; and H3: National savings rates impact MPI scores.

This study not only investigates the impact of national savings on economic development in Sub-Saharan Africa but also examines the interplay of inflation rates and foreign direct investments, aiming to provide strategic recommendations for policymakers to foster national savings and catalyze socio-economic transformation in the region. Through its comprehensive approach, the research contributes to the existing body of literature by uniquely integrating HDI, iHDI, and MPI as indicators of economic development, offering a nuanced understanding of the critical role of savings in driving sustainable development and poverty reduction in Sub-Saharan Africa.

### Scope and rationale

To test the hypothesis, the population of interest in this study is Sub-Saharan Africa, which is uniquely characterized by the highest prevalence of poverty globally. To provide a true representation of the economic development situation in the continent, ten countries with the highest number of poor individuals are selected: Nigeria, with 86.9 million poor; D.R. Congo, with 60.9 million; Ethiopia, with 23.9 million; Tanzania, with 19.9 million; Mozambique, with 17.8 million; Kenya, with 14.7 million; Uganda, with 14.2 million; South Africa, with 13.8 million; South Sudan, with 11.4 million; and Zambia, with 9.5 million. These ten countries account for 13 out of the 15 nations worldwide where extreme poverty is on the rise^15^. Cross-section data from the World Bank, the UNDP, and OPHD for the selected countries reveal significant insights into the region’s savings and investment landscape. The mean national savings rate across the ten countries stands at 17.85%, highlighting the variation in savings behaviors and their potential impact on economic growth. The Human Development Index (HDI), which provides a composite measure of life expectancy, education, and per capita income, shows an average score of 0.509, reflecting varied levels of development across these nations. The Multidimensional Poverty Index (MPI), with an average value of 0.344, underscores the complex challenges related to poverty that continue to impede economic development. Lastly, the Inequality-adjusted Human Development Index (iHDI), averaging at 0.345, further elucidates the disparities in human development outcomes within these countries.Sequqntial Generalized Method of Moments (GMM) is employed in the analysis of the impact of National savings on the three (3) economic development indicators. The use of sequential GMM allows for the estimation of dynamic panel data models while addressing potential endogeneity issues.

### Structure of the paper

The paper is organized as follows: Following the Introduction, the Literature Review section discusses theoretical frameworks and previous empirical studies. The Methodology section describes the econometric models and data sources used. The Empirical Results section presents the findings, followed by Conclusions and Policy Recommendations, which highlight the implications of the study and suggest avenues for future research.

## Literature review

Savings are conceptualized as deferred consumption and are essential for economic growth. Gross national savings, comprising individual, business, and government savings, significantly influence a country’s GDP and wealth creation^16,17^.

### Theoretical frameworks

The relationship between national savings and economic development is explored through several theoretical models. Rostow’s Stages of Growth, the Harrod–Domar Growth Model, and the Solow Growth Model offer diverse insights into this relationship. Rostow links stages of economic development to savings and investment (Rostow 1950s–1960s). The Harrod-Domar model emphasizes the role of savings in capital accumulation and its impact on economic growth, particularly in developing countries with labor surplus^5,6^. The Solow Model explores output changes due to varying savings rates, population growth, and technological progress, highlighting the role of savings in long-term economic growth^7^.

Nordhaus^8^ argued that insufficient savings lead to declines in economic development and living standards. He suggested that increased savings would result in higher investment, boosting output, real wages, and living standards. However, he also acknowledged Keynes’ concern that increased savings might reduce aggregate demand and increase short-term unemployment. Strategies to increase national savings include income-affecting policies like tax adjustments and price-affecting policies that incentivize savings^8^. Effective channeling of increased savings into high-priority investments is crucial for sustainable economic development.

Economic growth, primarily concerned with output increase, differs from economic development, which encompasses improvements in quality of life, innovation, and social welfare^9,18^. Traditional GDP measures are insufficient to capture the multifaceted nature of economic development. Alternative measures like the Human Development Index (HDI), Inequality-adjusted Human Development Index (iHDI), and Multidimensional Poverty Index (MPI) provide a more comprehensive understanding of economic development^19^. The HDI reflects three key dimensions of human development: life expectancy at birth, education levels, and income. It aggregates quantifiable indicators but does not incorporate essential subjective and qualitative aspects of human development. The iHDI adjusts the HDI for inequality in the distribution of each dimension across the population, providing insights into policies aimed at reducing inequalities and understanding how disparities impact overall human development. The MPI identifies the most vulnerable populations, revealing poverty patterns within countries over time, and allowing policymakers to allocate resources and design policies more effectively.

### Empirical evidence

National savings, defined as the total of gross national income minus total consumption plus net transfers, represents the total savings generated within a country from both the public and private sectors. It is essential for funding investments that spur economic growth. Empirical evidence suggests that increasing the national savings rate can contribute to economic growth. Iqbal et al.^20^ found that the national savings rate and credit extended to the private sector played a significant role in Pakistan’s economic growth and development, with a 1% increase in the national savings rate leading to a 1.015% increase in GDP growth. Ciftcioglu and Begovic^21^ used panel data analysis to examine the relationship between the savings rate and economic growth in Central and Eastern European countries, finding a statistically significant impact of domestic savings rate on GDP growth. Akinola and Omolade^22^ investigated the effects of the national savings rate and capital stock development on economic growth in Nigeria, demonstrating their contribution to economic growth and capital accumulation.

However, Griffin^23^ argued that economic growth in many developing countries has not effectively reduced poverty, suggesting that the resources required for development are often not properly utilized. Bishop and Cassidy^24^ reported that Australia’s high household, corporate, and national savings rates have funded investments in the mining sector and reduced current account deficits. They noted that reducing budget deficits can increase the national savings rate and improve future living standards.

Reviewed studies consistently underscore the critical role of savings in facilitating investment and fostering economic growth. However, the mechanisms through which savings rates influence specific socio-economic outcomes, such as human development and poverty reduction, remains excluded or had received a muted discussion.

Based on the theoretical foundations and empirical evidence discussed, this study hypothesizes that higher national savings rates impact economic development in Sub-Saharan Africa, as measured by the Human Development Index (HDI), Inequality-adjusted HDI (iHDI), and Multidimensional Poverty Index (MPI). Specifically, we expect that: H1: National savings rates impact HDI scores.; H2: National savings rates impact iHDI scores.; and H3: National savings rates impact MPI scores.

### Gaps in the literature

Identification of research gaps, particularly in the context of the poorest countries in the Sub-Saharan Africa, such as limited studies on the direct causal relationships between national savings and specific dimensions of economic development and the need for more comprehensive analyses that include institutional quality as a variable.

This study contributes to the existing literature on the relationship between national savings and economic development in several ways. First, it focuses on Sub-Saharan Africa, a region that has received relatively less attention in the savings-growth literature compared to other developing regions. Second, it employs a comprehensive set of economic development indicators (HDI, iHDI, and MPI) to capture the multidimensional nature of economic progress, going beyond traditional measures such as GDP growth. Third, it utilizes panel data analysis to account for both cross-country and time-series variations in the relationship between savings and development. By addressing these gaps in the current research, this study aims to provide new insights into the role of savings in promoting sustainable economic development in Sub-Saharan Africa.

## Methodology

### Econometric model

The study's empirical model specification draws from several theoretical frameworks and empirical studies that highlight the importance of savings in economic growth and development. These include Rostow’s Stages of Growth model, the Harrod–Domar Growth Model, and the Solow Growth Model, which emphasize the role of savings in capital accumulation, investment, and long-term economic growth. Empirical evidence from Nordhaus^8^ further supports the link between increased savings, higher investment, output, real wages, and living standards.The study also recognizes the distinction between economic growth and economic development, with the latter encompassing improvements in quality of life, innovation, and social welfare.

By incorporating these theoretical foundations and empirical evidence, the study aims to provide a more comprehensive understanding of the drivers of economic development in sub-Saharan Africa, while acknowledging the limitations of traditional GDP measures and the need for alternative indicators like the HDI, iHDI, and MPI.

The econometric models that will be estimated are as follows:

1 $$\begin{aligned} \ln HDI_{it} & = \beta_{0} + \beta_{1} \ln HDI_{it - 1} + \beta_{2} \ln NatSav_{it} + \beta_{3} \ln GOVEX_{it} + \beta_{4} \ln INF_{it} \\ & \quad + \beta_{5} \ln ODA_{it} + \beta_{6} \ln FDI_{it} + \beta_{7} \ln Comp1_{it} + \beta_{8} \ln Comp2_{it} + u_{it} \\ \end{aligned}$$

2 $$\begin{aligned} \ln iHDI_{it} & = \beta_{0} + \beta_{1} \ln iHDI_{it - 1} + \beta_{2} \ln NatSav_{it} + \beta_{3} \ln GOVEX_{it} + \beta_{4} \ln INF_{it} \\ & \quad + \beta_{5} \ln ODA_{it} + \beta_{6} \ln FDI_{it} + \beta_{7} \ln Comp1_{it} + \beta_{8} \ln Comp2_{it} + u_{it} \\ \end{aligned}$$

3 $$\begin{aligned} \ln MPI_{it} & = \beta_{0} + \beta_{1} \ln MPI_{it - 1} + \beta_{2} \ln NatSav_{it} + \beta_{3} \ln GOVEX_{it} + \beta_{4} \ln INF_{it} \\ & \quad + \beta_{5} \ln ODA_{it} + \beta_{6} \ln FDI_{it} + \beta_{7} \ln Comp1_{it} + \beta_{8} \ln Comp2_{it} + u_{it} \\ \end{aligned}$$

where *lnHDI* is the of Log of Human Development Index, *lniHDI* is the Log of inequality-adjusted human development index, *lnMPI* is the Log of multidimensional poverty index, *lnNatSav* is the Log of national savings rate, *lnGOVEX* is Log of Government expenditure, *lnINF* is Log of Inflation, *lnODA* is Log of Official Development Assistance, *lnFDI* is Log of Foreign Direct Investment, *lnComp1* and *lncomp2* represents the log of institutional quality obtained by PCA on the six governance indicators (Table 1) and U is stochastic error term.

**Table 1:** Principal component analysis. *Source*: Authors’ computation.

Principal components/correlation	Number of obs = 97			
	Number of comp. = 6			
	Trace = 6			
Rotation: (unrotated = principal)	Rho = 1.0000			

Component	Eigenvalue	Difference	Proportion	Cumulative
Comp1	4.85435	4.35941	0.8091	0.8091
Comp2	0.494941	0.111567	0.0825	0.8915
Comp3	0.383374	0.201267	0.0639	0.9554
Comp4	0.182108	0.124113	0.0304	0.9858
Comp5	0.0579945	0.0307644	0.0097	0.9955
Comp6	0.0272301	–	0.0045	1.0000

### Construction of institutional quality index

The institutional quality variable is represented by six indicators: (i) voice and accountability, which assesses citizens' ability to participate in government appointments; (ii) political stability/absence of violence, which reflects the government's inclination towards non-institutional methods and violence, including terrorism; (iii) regulatory quality, which gauges the government's capability to develop and enforce effective policies and regulations; (iv) government effectiveness, which evaluates the quality of public service delivery, the competence of public administration and officials, the impartiality of civil services from political pressures, and the credibility of public authorities in their commitments and policies; (v) rule of law, which measures the state's ability to uphold and enforce contracts; and (vi) control of corruption, which indicates the level of corruption and the misuse of public power for private gain^25,26^.

Moreover, using multiple institutional indicators in modeling can lead to multicollinearity issues, as these indicators are highly correlated with each other^27,28^. Therefore, this study began with six governance indicators to create a synthetic index through principal component analysis (PCA).

The first component (Comp1) has an eigenvalue of 4.85435, which explains approximately 80.91% of the total variance. This high proportion indicates that Comp1 captures the majority of the information contained in the six governance indicators. The substantial eigenvalue difference between Comp1 and Comp2 (4.35941) further underscores the dominant role of the first component. The PCA technique allowed this study to retain the first component in accordance with the criterion of Kaiser^29^ and Jolliffe^30^. This component has an eigenvalue greater than 1 and represents 80.91% of the data variation and will be used as a measure for institutional quality.

### Data and source

This study employs a panel data analysis covering the period from 2009 to 2019, focusing on ten of the poorest countries in Sub-Saharan Africa. The selection of this time frame and these specific countries is grounded in several key considerations:

- The period from 2009 to 2019 encompasses significant economic transitions and policy shifts in Sub-Saharan Africa, offering a comprehensive view of the evolving economic landscape. This timeframe allows for the analysis of long-term trends in national savings and economic development, including the impact of the global financial crisis of 2008 and subsequent recovery efforts. Additionally, recent data up to 2019 provides insights into the preliminary effects of the COVID-19 pandemic on national savings and economic resilience.
- The countries selected for this study are among the poorest in Sub-Saharan Africa, as determined by their Gross National Income (GNI) per capita and Human Development Index (HDI) scores. This focus on the poorest countries is deliberate, as these nations face unique challenges in mobilizing domestic savings for investment and development. By examining these countries, the study aims to shed light on the potential of national savings as a lever for economic improvement in contexts characterized by significant developmental hurdles.

The study uses a range of socio-economic indicators, including the Human Development Index (HDI), Inequality-adjusted HDI (iHDI), and Multidimensional Poverty Index (MPI), to assess the impact of national savings and other control variables on development outcomes (Table 2). These indicators were chosen for their comprehensive impact coverage of human development and poverty dimensions, allowing for a nuanced analysis of the savings-development nexus. Data sources include reputable international organizations such as the World Bank, United Nations Development Programme (UNDP), and International Monetary Fund (IMF), ensuring the reliability and comparability of the data.

**Table 2:** Estimation variables description. *Source*: Author.

Variable	Measures	Sources	Signs
HDI	Human Development Index, a composite measure comprising life expectancy at birth, education, and incomes. It does not consider fundamental subjective and qualitative components	UNDP	+
iHDI	Inequality-adjusted Human Development Index, which accounts for inequalities in dimensions, informing policies towards inequality reduction and better understanding of inequalities across populations. It includes the Coefficient of Human Inequality, calculated as an unweighted average of inequality across three dimensions	UNDP	+
MPI	Multidimensional Poverty Index, identifying the most vulnerable individuals among the poor (bottom 40% of the poor population), revealing poverty patterns within countries over time, and enabling policymakers to target resources and design policies more effectively	UNDP/OPHI	+
NatSav	Gross savings, calculated as gross national income minus total consumption, plus net transfers	World Bank	+
GOVEX	General government final consumption expenditure, including all government current expenditures for goods and services acquisition, employee compensation, and most national defense and security expenditures	World Bank	+/−
INF	Quantitative measure of the rate at which the average price level of a 'basket' of selected goods and services in an economy rises over a period. Expressed as a percentage, indicating a decrease in the purchasing power of the currency	World Bank	−
ODA	Payments of loans on concessional terms (net of principal repayments) and grants by official agencies of DAC members, multilateral institutions, and non-DAC countries to promote economic development and welfare in DAC-listed ODA recipient countries. Includes loans with at least a 25% grant element (calculated at a 10% discount rate)	OECD	+
FDI	Net inflows of investment to acquire a lasting management interest (10% or more of voting stock) in an enterprise operating in an economy other than that of the investor. Includes equity capital, reinvested earnings, other long-term capital, and short-term capital as shown in the balance of payments. Data shows net inflows (new investment inflows less disinvestment) divided by GDP	World Bank	+
Comp1	Institutional quality, the first principle component of voice and accountability; political stability/absence of violence; regulatory quality government; effectiveness; rule of law; and control of corruption	WDI	+/−
Comp2	Institutional quality, the second principle component of voice and accountability; political stability/absence of violence; regulatory quality government; effectiveness; rule of law; and control of corruption	WDI	+/−

UNDP stands for United Nations Development Programme, OPHI stands for Oxford Poverty and Human Development Initiative, WDI stands for World Development Indicators, and OECD stands for Organisation for Economic Co-operation and Development.

The study uses a panel dataset of the 10 poorest sub-Saharan African countries (Nigeria, D.R. Congo, Ethiopia, Tanzania, Mozambique, Kenya, Uganda, South Africa, South Sudan, and Zambia) over the period 2009–2019. The countries were chosen based on the number of poor people in each country, with the 10 countries representing the countries with the highest number of poor individuals in the region.

For every variable in each of the countries, the descriptive analysis (Table 3) shows that from 2009 to 2019:

**Table 3:** Descriptive statistic. *Source*: Author.

Country	Indicator	Count	Mean	Std Dev	Min	Max
Ethiopia	mpi	8	0.55	0.03	0.49	0.56
	hdi	9	0.44	0.02	0.40	0.46
	ihdi	8	0.31	0.03	0.27	0.35
	natsav	7	30.67	1.50	28.33	33.08
	govex	8	9.79	1.06	8.30	11.13
	inf	9	12.97	9.25	7.26	33.25
	oda	9	8.01	2.79	5.03	11.80
	fdi	10	2.87	1.74	0.64	5.37
	comp1	10	− 0.85	0.23	− 1.12	− 0.45
Kenya	mpi	8	0.22	0.04	0.18	0.30
	hdi	9	0.56	0.02	0.53	0.59
	ihdi	8	0.39	0.03	0.37	0.43
	natsav	9	12.12	1.73	9.61	14.51
	govex	10	13.79	0.75	12.71	15.21
	inf	10	7.48	2.90	3.96	14.02
	oda	9	4.53	1.07	3.17	6.07
	fdi	10	1.46	1.05	0.31	3.46
	comp1	10	0.21	0.34	− 0.38	0.57
Mozambique	mpi	8	0.43	0.06	0.39	0.51
	hdi	9	0.42	0.01	0.40	0.44
	ihdi	8	0.28	0.03	0.22	0.30
	natsav	10	7.76	2.96	2.04	10.86
	govex	10	22.39	3.48	17.48	26.47
	inf	0				
	oda	9	14.10	2.24	11.59	18.08
	fdi	10	23.30	9.81	7.82	39.46
	comp1	10	0.39	0.91	− 0.86	1.45
Nigeria	mpi	8	0.30	0.03	0.24	0.37
	hdi	9	0.51	0.02	0.48	0.53
	ihdi	8	0.32	0.02	0.28	0.35
	natsav	9	21.58	5.25	15.82	31.89
	govex	10	7.00	1.56	4.40	8.85
	inf	10	11.82	2.89	8.06	16.52
	oda	9	0.57	0.15	0.44	0.92
	fdi	10	1.33	0.75	0.50	2.93
	comp1	10	− 1.72	0.22	− 2.01	− 1.42
South Africa	mpi	8	0.04	0.02	0.01	0.06
	hdi	9	0.67	0.02	0.64	0.70
	ihdi	5	0.45	0.01	0.44	0.47
	natsav	10	16.32	1.09	14.88	18.01
	govex	10	20.49	0.45	19.86	21.28
	inf	10	5.48	1.01	4.06	7.26
	oda	9	0.35	0.06	0.28	0.46
	fdi	10	1.29	0.70	0.48	2.58
	comp1	10	3.68	0.36	3.04	4.10
South Sudan	mpi	4	0.56	0.01	0.56	0.58
	hdi	8	0.40	0.01	0.39	0.42
	ihdi	1	0.25		0.25	0.25
	natsav	3	− 6.31	2.66	− 9.38	− 4.73
	govex	7	26.30	23.81	12.62	79.17
	inf	9	80.08	126.96	− 0.04	379.85
	oda	5	10.89	4.60	5.10	15.71
	fdi	4	− 0.74	2.46	− 4.30	1.35
	comp1	8	− 4.84	1.26	− 6.13	− 3.08
Tanzania	mpi	8	0.32	0.03	0.27	0.37
	hdi	9	0.51	0.02	0.49	0.54
	ihdi	8	0.38	0.02	0.35	0.40
	natsav	9	26.81	2.41	22.58	30.54
	govex	9	10.02	1.06	8.50	12.33
	inf	10	8.06	4.10	3.49	16.00
	oda	9	7.00	2.09	4.76	10.86
	fdi	10	3.52	1.14	1.90	5.66
	comp1	10	0.87	0.23	0.44	1.23
Uganda	mpi	8	0.36	0.03	0.28	0.37
	hdi	9	0.50	0.01	0.48	0.52
	ihdi	8	0.34	0.02	0.33	0.37
	natsav	10	19.05	1.15	17.09	21.06
	govex	10	9.42	1.73	7.50	12.79
	inf	10	7.33	4.87	2.62	16.56
	oda	9	7.59	1.21	6.12	10.00
	fdi	10	3.86	1.00	2.59	5.21
	comp1	10	0.33	0.09	0.21	0.48
Zambia	mpi	7	0.30	0.03	0.26	0.33
	hdi	9	0.57	0.02	0.53	0.59
	ihdi	8	0.38	0.01	0.36	0.39
	natsav	7	32.68	1.79	30.68	35.86
	govex	7	12.73	2.48	9.38	16.07
	inf	10	9.17	3.73	6.43	17.87
	oda	9	4.72	1.49	3.75	8.52
	fdi	10	5.31	2.25	1.53	8.53
	comp1	10	1.37	0.33	0.94	1.86
Democratic Republic of Congo	mpi	8	0.39	0.01	0.38	0.40
	hdi	9	0.43	0.02	0.40	0.46
	ihdi	8	0.28	0.03	0.25	0.32
	natsav	10	15.07	5.14	8.16	21.65
	govex	10	7.68	0.99	6.07	9.06
	inf	8	5.08	5.24	0.74	15.32
	oda	9	10.91	5.65	5.78	22.52
	fdi	10	4.79	4.01	− 1.30	12.72
	comp1	0				

For Ethiopia, the MPI has a mean of 0.555, indicating a moderate level of multidimensional poverty, while the HDI stands at 0.436, reflecting low human development. The IHDI averages 0.310, accounting for inequality impacts. National savings average 30.67% of GDP, with government expenditure at 9.79%. The inflation rate is high, averaging 12.97%, peaking at 33.25%. Official Development Assistance averages 8.01% of GDP, and FDI inflows average 2.87%. In Kenya, the MPI mean is 0.222, suggesting lower multidimensional poverty than Ethiopia. The HDI is higher at 0.519, with an IHDI of 0.376. National savings are lower, averaging 11.78%, while government expenditure is at 11.45%. The inflation rate averages 8.50%, with ODA at 4.88% and FDI at 0.92%. The composite index, comp1, averages − 0.85. Mozambique exhibits a higher MPI mean of 0.364, indicating greater multidimensional poverty, and an HDI of 0.393. The IHDI is 0.267, and national savings average 14.29%. Government expenditure stands at 11.08%, with inflation averaging 7.52%. ODA is significant at 22.58%, while FDI averages 3.56%. The comp1 index for Mozambique is not available. Nigeria's MPI mean is 0.344, with an HDI of 0.510 and an IHDI of 0.293. National savings are at 23.12%, and government expenditure averages 8.34%. The inflation rate is 12.36%, ODA is at 0.46%, and FDI inflows average 1.95%. The comp1 index averages − 1.82. South Africa has the highest HDI mean at 0.700, though MPI data is not available. The IHDI averages 0.520. National savings stand at 16.74%, with government expenditure significantly higher at 20.91%. The inflation rate averages 5.59%, ODA is at 0.30%, and FDI inflows average 1.21%. The comp1 index averages − 0.36. South Sudan’s HDI mean is 0.433, with MPI and IHDI data not available. National savings average 9.23%, and government expenditure is 12.39%. The country experiences severe economic instability, with an average inflation rate of 104.13%. ODA averages 27.55%, while FDI inflows average 3.20%. The comp1 index for South Sudan is not available. In Tanzania, the MPI mean is 0.284, with an HDI of 0.488 and an IHDI of 0.350. National savings average 22.17%, with government expenditure at 14.92%. The inflation rate averages 7.69%, ODA is at 11.39%, and FDI inflows average 3.53%. The comp1 index averages − 1.53. Uganda shows an MPI mean of 0.284, an HDI of 0.493, and an IHDI of 0.357. National savings average 17.59%, with government expenditure at 12.38%. The inflation rate averages 6.76%, ODA is at 11.99%, and FDI inflows average 3.87%. The comp1 index averages − 1.40. Zambia’s MPI mean is 0.298, with an HDI of 0.568 and an IHDI of 0.379. National savings average 32.68%, and government expenditure is 12.73%. The inflation rate averages 9.17%, ODA is at 4.72%, and FDI inflows average 5.31%. The comp1 index averages 1.37. The Democratic Republic of Congo has an MPI mean of 0.367, an HDI of 0.457, with IHDI data not available. National savings average 3.40%, government expenditure is 7.68%, and the inflation rate averages 5.08%. ODA averages 10.91%, while FDI inflows average 4.79%. The comp1 index for the Democratic Republic of Congo is not available. These statistics provide a snapshot of the socio-economic conditions in each country, highlighting variations in poverty, development, savings, government spending, inflation, and external financial support.

The analysis of socio-economic indicators across the ten countries reveals significant variations in development, economic stability, and external support. For the period under review, The Democratic Republic of Congo has the highest mean MPI at 0.367, indicating severe multidimensional poverty, while Kenya has the lowest mean MPI at 0.222, reflecting better conditions in this regard. South Africa boasts the highest mean HDI of 0.700, signifying a higher level of human development. In contrast, Mozambique has the lowest mean HDI at 0.393, indicating lower human development. Zambia leads with the highest mean IHDI of 0.379, suggesting better adjusted human development levels. Mozambique has the lowest mean IHDI at 0.267, reflecting significant inequality impacts. Zambia again stands out with the highest mean national savings at 32.68% of GDP, indicating strong savings habits or policies. The Democratic Republic of Congo has the lowest mean at 3.40%, suggesting weaker savings. South Africa has the highest mean government expenditure at 20.91% of GDP, pointing to significant public sector investment. The Democratic Republic of Congo shows the lowest mean at 7.68%, indicating limited government spending. South Sudan has the highest mean inflation rate at a staggering 104.13%, highlighting extreme economic instability. The Democratic Republic of Congo has the lowest mean inflation rate at 5.08%, indicating relatively stable prices. South Sudan receives the highest mean ODA at 27.55% of GDP, reflecting heavy reliance on external aid. Nigeria receives the least mean ODA at 0.46%, indicating lesser dependence on such assistance. Zambia attracts the highest mean FDI inflows at 5.31% of GDP, showing strong foreign investment interest. Kenya has the lowest mean FDI at 0.92%, indicating lesser foreign investment. Zambia has the highest mean institutional quality composite index at 1.37, which can signify better overall conditions according to the metrics used in this index. Overall, these statistics provide a clear picture of where each country stands in terms of development, economic policies, and external support. South Africa and Zambia generally show better socio-economic conditions, while South Sudan and the Democratic Republic of Congo face significant challenges (within the period 2009–2019).

### Econometric methods and procedures

To address potential endogeneity issues, the analysis will employ the Generalized Method of Moments (GMM), a statistical method that provides robust estimations by using moment conditions derived from the data. GMM is particularly advantageous because it can handle models with endogenous variables, allows for efficient estimation even when there is heteroskedasticity or autocorrelation in the error terms, and can accommodate dynamic panel data models^31^.

Log-transformed variables are used in the analysis to account for the non-linearity of the data and to ensure standardized distributions. This approach will also allow the results to be interpreted in terms of elasticities, facilitating comparisons with other studies.

Additionally, the Augmented Dickey–Fuller (ADF) test will be applied to test for stationarity in the data^32^. Ensuring that the variables are stationary is crucial for the reliability of the GMM estimators. To test for stationarity in the data, the Augmented Dickey–Fuller (ADF) test will be applied. The ADF test is a common method for detecting the presence of a unit root in a time series sample, which indicates non-stationarity^32^. The presence of a unit root implies that shocks to the time series can have permanent effects, making the series unpredictable in the long run.

Also, it is particularly important because it helps in providing a more accurate test statistic by adjusting for potential autocorrelation in the error terms. The null hypothesis of the ADF test states that (gamma = 0), indicating a unit root is present (i.e., the time series is non-stationary). If the null hypothesis is rejected, it suggests that the time series does not have a unit root and is stationary. By employing the ADF test, we can ensure that the variables used in the GMM estimation are stationary, thus avoiding the issues of spurious regression results. This step is crucial because non-stationary data can lead to unreliable and misleading inferences, which would undermine the validity of the econometric analysis. Furthermore, verifying stationarity helps in achieving more robust and interpretable results, which is essential for policy implications and for comparing outcomes across different studies.

## Result and analysis

### Panel unit root test

The Fisher-type unit-root test based on Augmented Dickey–Fuller (ADF) tests was conducted to assess the stationarity of the independent variables used in the analysis. Stationarity is a crucial assumption for reliable and valid results in econometric models, particularly for the Generalized Method of Moments (GMM) estimation. The results of the test for each independent variable are summarized in Table 4.

**Table 4:** Fisher-type unit-root test based on Augmented Dickey–Fuller (ADF) tests. *Source*: Author.

Independent variables	Results
lnHDI	0.0002***
lniHDI	0.0000***
lnMPI	0.0001***
lnNatSav	0.0000***
lnGOVEX	0.0000***
lnINF	0.0000***
lnODA	0.0000***
lnFDI	0.0080***
lnComp1	0.0376**

Significance Levels *** (*p* < 0.01), ** (*p* < 0.05).

The table presents the p-values for each independent variable, indicating whether we can reject the null hypothesis that all panels contain unit roots (i.e., the series are non-stationary) in favor of the alternative hypothesis that at least one panel is stationary. The significance levels are denoted as follows: (*p* < 0.01), (*p* < 0.05). lnHDI (Human Development Index) with the *p*-value of 0.0002 indicates a high level of significance (*p* < 0.01), allowing us to reject the null hypothesis and conclude that the panel data for lnHDI is stationary. lniHDI (Income Human Development Index) With a *p*-value of 0.0000, this variable is highly significant (*p* < 0.01), suggesting that the data is stationary. lnMPI (Multidimensional Poverty Index) with the p-value of 0.0001 shows high significance (*p* < 0.01), confirming the stationarity of this variable. lnNatSav (National Savings) with the *p*-value of 0.0000 indicates strong evidence (*p* < 0.01) that the panel data is stationary. lnGOVEX (Government Expenditure) with A *p*-value of 0.0000 demonstrates high significance (*p* < 0.01), indicating the data is stationary. lnINF (Inflation) With a *p*-value of 0.0000, the data is highly significant (*p* < 0.01), confirming its stationarity. lnODA (Official Development Assistance) with the *p*-value of 0.0000 shows high significance (*p* < 0.01), suggesting the data is stationary. lnFDI (Foreign Direct Investment) with the *p*-value of 0.0080 indicates significance at the 1% level (*p* < 0.01), confirming the stationarity of the data. lnComp1 with the p-value of 0.0376 is significant at the 5% level (*p* < 0.05), suggesting that the panel data for lnComp1 is stationary.

The results of the Fisher-type unit-root test are favorable for the application of the Generalized Method of Moments (GMM) in this analysis. Since all the independent variables exhibit stationarity, the risk of spurious regression is minimized. This enhances the reliability of the GMM estimators, as the method relies on the assumption of stationary series to provide robust and consistent estimates. The presence of stationary data ensures that the moment conditions used in GMM are valid, thereby improving the efficiency and accuracy of the estimations. Overall, the significant *p*-values across all variables confirm the appropriateness of using GMM for this econometric analysis. The robustness of the GMM estimators in handling heteroskedasticity and autocorrelation is further supported by the stationarity of the panel data, ensuring that the results are both reliable and interpretable.

### Estimation results and discussion

The choice for the GMM approach is driven by its robustness in addressing potential endogeneity issues. Furthermore, GMM is particularly advantageous in handling heteroskedasticity and serial autocorrelation, providing more reliable and consistent parameter estimates even in the presence of these issues^33^. Additionally, this study employed sequential analysis in the estimation process. This method involves analyzing data in a step-by-step manner, allowing for interim analyses and decisions to be made as the data analysis progresses. The primary advantage of sequential analysis is its flexibility and efficiency. It enables the identification of trends or impact patterns early on, potentially saving time (for analysis and interpretation) without compromising the validity of the results. By leveraging GMM and sequential analysis, the estimation process becomes both rigorous and adaptive, ensuring that the results are robust and reflective of the underlying economic phenomena.

In Table 5 below, the sequential GMM analysis conducted in this study provides valuable insights into the factors influencing the Human Development Index (HDI) in Sub-Saharan African countries. The results from the seven models highlight the complex relationships between national savings, government expenditure, inflation, official development assistance, foreign direct investment, and institutional quality on human development. Model 1, which examines the relationship between national savings and HDI, reveals a significant positive impact of lagged HDI (L.lnhdii) on current HDI, with a coefficient of 0.824 and a highly significant *p*-value (*p* < 0.001). This indicates that the HDI of the previous period positively influences the current HDI, suggesting a persistence in human development levels over time. However, the negative impact of national savings (lnnatsavi) observed in this study, with a coefficient of − 0.0566 (though not significant), suggests that in the context of Sub-Saharan Africa, national savings may not be effectively utilized for productive or welfare-enhancing activities. This finding aligns with the theoretical models proposed by Harrod^6^ and Domar^5^, which emphasize the role of savings in capital accumulation and its impact on economic growth, especially in developing countries with labor surplus. Additionally, empirical studies by Adebiyi^1^ and Ciftcioglu and Begovic^21^ support the notion that efficient financial intermediation is crucial for translating savings into economic growth and development. Model 2 introduces government expenditure (lngovexi) as an additional variable, revealing a positive and significant impact on HDI with a coefficient of 0.158 (*p* < 0.001). This result is consistent with the findings of Olasehinde-Williams^16^, who argues that government expenditure on social services such as education and healthcare can significantly enhance human development. The negative coefficient for national savings remains, reinforcing the need for effective utilization of savings in productive investments. Model 3 incorporates inflation (lninfi) into the analysis, showing a positive but not statistically significant impact on HDI with a coefficient of 0.00589. This mixed result aligns with the findings of Budhedeo^4^, who highlights the complex relationship between inflation and economic growth, suggesting that moderate inflation may stimulate growth, while high inflation can be detrimental. Model 4 includes official development assistance (lnodai), which shows a negative impact on HDI with a coefficient of − 0.042. This finding is supported by Griffin^23^, who argues that foreign aid may not always lead to positive development outcomes due to issues such as dependency and misallocation of resources. Model 5 examines the role of foreign direct investment (lnfdii), revealing a positive but not statistically significant impact on HDI with a cofficient of 0.0267. This result is consistent with the theoretical perspectives of Rostow^34^ and empirical studies by Akinola and Omolade^22^, which suggest that FDI can contribute to economic growth and development by bringing in capital, technology, and managerial expertise. Model 6 introduces composite indices of institutional quality, lncomp1 showing a negative significant impact on HDI with a coefficient of − 0.000536. This finding aligns with the arguments of Feldman et al.^9^, who emphasize the importance of good governance and institutional quality in promoting sustainable development. The negative coefficients suggest that poor institutional quality may hinder the effective utilization of resources for human development. Model 7 combines all the variables (including comp2 which is not significant), with the results indicating that government expenditure and FDI are positively associated with human development, while higher national savings and the composite indices of institutional quality might have a negative association. The role of inflation and official development assistance remains mixed. The coefficient for the lagged HDI is 0.293, though it is not statistically significant (*p* = 0.568). This suggests that past HDI levels do not have a substantial predictive power for current HDI levels within this model. This lack of significance could imply that other contemporaneous factors might be more influential in determining current HDI. The coefficient for national savings is − 0.0862, which is not statistically significant (*p* = 0.0443). Despite its lack of statistical significance, the negative sign indicates a potential inverse relationship where higher national savings may be associated with a lower HDI. This finding aligns with the notion that savings, if not effectively channeled into productive investments, may not directly enhance human development^1^. The coefficient for government expenditure is 0.101, but it is not statistically significant (*p* = 0.119). This suggests that within this model, government expenditure does not have a significant direct impact on HDI. However, effective government expenditure remains essential for human development through its potential indirect effects on health, education, and infrastructure^9^. Inflation has a positive coefficient of 0.00520 and is statistically significant at the 5% level (*p* < 0.05). This indicates that moderate inflation may have a positive impact on HDI, possibly through stimulating economic activity and increasing incomes, which can then improve human development outcomes^10^. The coefficient for official development assistance is − 0.0399, which is statistically significant (*p* < 0.01). This negative relationship suggests that higher levels of ODA are associated with lower HDI. This could be due to the inefficiencies and dependencies that aid might create, rather than fostering sustainable development^12^. The coefficient for FDI is 0.0208, although it is not statistically significant (*p* = 0.0224). The positive sign suggests that FDI might contribute to improvements in HDI by bringing in capital, technology, and expertise that enhance economic activities and living standards^13^. The coefficient for the first institutional quality composite index is − 0.000513, which is statistically significant at the 1% level (*p* < 0.001). This negative relationship suggests that higher institutional quality, as measured by this index, is associated with a lower HDI in the short term. This could reflect the transitional costs associated with institutional reforms before their benefits are realized^11^. The coefficient for the second institutional quality composite index is − 0.000135, but it is not statistically significant (*p* = 0.207). This indicates that the second measure of institutional quality does not have a significant impact on HDI within this model. Overall, the results suggest that government expenditure and FDI are positively associated with human development, while higher national savings and the composite indices of institutional quality might have a negative association. The role of inflation and official development assistance is mixed. These findings highlight the importance of effective utilization of savings, government expenditure, and foreign investment in promoting human development, while also suggesting the need for a deeper understanding of the components of institutional quality and their impact on HDI. This study contributes to the literature by offering a comprehensive analysis of the savings-development nexus in Sub-Saharan Africa, integrating the HDI, iHDI, and MPI as measures of socio-economic progress, as hypothesized by Rostow^34^, Harrod^6^, Domar^5^ and Solow^7^.

**Table 5:** Sequential GMM estimation results of National Savings and HDI. *Source*: Author.

	(1) lnhdii	(2) lnhdii	(3) lnhdii	(4) lnhdii	(5) lnhdii	(6) lnhdii	(7) lnhdii
esttab modell model2 model3 mode14 model5 model6 model7, se stats(11 chi2)							
L.lnhdii	0.824*** (8.52e−10)	0.235 (·)	0.200*** (9.09e−09)	0.396 (·)	− 0.0267 (·)	0.118 (0.245)	0.293 (0.568)
lnnatsavi	− 0.0566 (·)	− 0.0706 (·)	− 0.0654*** (3.56e−10)	− 0.0578 (·)	− 0.105*** (3.48e−10)	− 0.0943*** (0.0169)	− 0.0862 (0.0443)
lngovexi		0.177 (·)	0.158*** (2.97e−09)	0.117 (·)	0.178 (·)	0.152** (0.0572)	0.101 (0.119)
lninfi			0.00589*** (1.88e−11)	0.00804 (·)	0.00472*** (4.96e−11)	0.00513*** (0.000983)	0.00520* (0.00264)
lnodai				− 0.0420 (·)	− 0.0396*** (2.12e−11)	− 0.0390*** (0.000498)	− 0.0399*** (0.00420)
lnfdii					0.0267 (·)	0.0204** (0.00695)	0.0208 (0.0224)
lncompl						− 0.000536** (0.000177)	− 0.000513*** (0.000114)
lncomp2							− 0.000135 (0.000207)
cons	0.0392 (·)	− 0.782 (·)	− 0.786*** (1.24e−08)	− 0.495 (·)	− 0.840 (·)	− 0.699* (0.276)	− 0.468 (0.589)
ll chi 2	9.35022e+17	0	4.03656e+15	0	9.16831e+16	510296.2	643.2

Standard errors in parentheses *p < 0.05, **p < 0.01, ***p < 0.001.

Table 6 below shows the results from the analysis for iHDI. Model 1, Lagged iHDI (L.lnihdii) has a coefficient of 1.114, but it is not statistically significant. This suggests that past iHDI levels do not significantly predict current iHDI in this model^35^. National Savings (lnnatsavi) has a coefficient is 0.00299, statistically significant (*p* < 0.001). This positive relationship indicates that higher national savings are associated with higher iHDI, emphasizing the importance of effective savings management^1^. In Model 2, Lagged iHDI (L.lnihdii) with a coefficient of 0.313, but not statistically significant, indicating limited predictive power of past iHDI on current levels^35^. National Savings (lnnatsavi) and Government Expenditure (lngovexi), No significance but with postive impact with coeeficients 0.0130 and 0.382 respectively. In Model 3, Lagged iHDI (L.lnihdii) has a coefficient of 0.219, not statistically significant^35^. National Savings (lnnatsavi) with a coefficient of 0.0103, statistically significant (*p* < 0.001), indicating a positive impact on iHDI^1^. Government Expenditure (lngovexi), The coefficient is 0.445, not statistically significant, suggesting government spending’s limited direct impact on iHDI here^9^. Inflation (lninfi), the coefficient is − 0.00367, statistically significant (*p* < 0.001), indicating negative effects of inflation on iHDI^10^. In Model Model 4, Lagged iHDI (L.lnihdii), The coefficient is − 0.579, not statistically significant^35^. National Savings (lnnatsavi), The coefficient is 0.00856, statistically significant (*p* < 0.001), reinforcing the positive impact of savings^1^. Government Expenditure (lngovexi), The coefficient is 0.803, not statistically significant^9^. Inflation (lninfi) and ODA (lnodai) with coefficients − 0.0128 (negative impact) and 0.0920 (positve impact) are both not significant. Model 5, Lagged iHDI (L.lnihdii), The coefficient is − 1.570, not statistically significant^35^. National Savings (lnnatsavi), The coefficient is − 0.0918, statistically significant (*p* < 0.001), indicating a negative relationship possibly due to inefficient use of savings^1^. Government Expenditure (lngovexi), The coefficient is 1.093, not statistically significant^9^. Inflation (lninfi), The coefficient is − 0.0303, not statistically significant^10^. ODA (lnodai), The coefficient is 0.155, not statistically significant^12^. FDI (lnfdii), The coefficient is 0.0730, statistically significant (*p* < 0.001), showing positive impact on iHDI witht savings^13^. Model 6, Lagged iHDI (L.lnihdii), The coefficient is − 1.528, statistically significant (*p* < 0.001), indicating negative predictive power^35^. National Savings (lnnatsavi), The coefficient is − 0.0917, statistically significant (*p* < 0.001), supporting the negative relationship observed in Model 5^1^. Government Expenditure (lngovexi), The coefficient is 1.070, statistically significant (*p* < 0.01), showing positive impact on iHDI^9^. Inflation (lninfi), The coefficient is − 0.0297, statistically significant (*p* < 0.01), indicating a negative impact on iHDI^10^. ODA (lnodai), The coefficient is 0.152, statistically significant (*p* < 0.01), showing a positive effect^12^. FDI (lnfdii), The coefficient is 0.0735, statistically significant (*p* < 0.001), highlighting the benefits of foreign investment and savings^13^. Institutional Quality Composite Index 1 (lncomp1), The coefficient is 0.000411, statistically significant (*p* < 0.05), indicating positive short-term effects^11^. with all the varibales n Model 7, The coefficient for the lagged iHDI is − 1.243, which is statistically significant at the 1% level (*p* < 0.001). This negative relationship suggests that past iHDI levels have a substantial negative predictive power for current iHDI levels within this model. This counterintuitive result might imply that past high levels of iHDI could be associated with diminishing returns in subsequent periods, possibly due to saturation effects where initial gains in human development are easier to achieve than subsequent ones^35^. The coefficient for national savings is − 0.0660, which is statistically significant (*p* < 0.01). This negative relationship indicates that higher national savings are associated with lower iHDI. This result could be indicative of the resource curse theory, where high levels of savings, particularly from natural resources, are not effectively channeled into productive investments that enhance human development^1^. This finding emphasizes the need for policies that ensure savings are invested in ways that directly benefit human development. The coefficient for government expenditure is 0.986, which is statistically significant at the 1% level (*p* < 0.01). This positive relationship underscores the critical role of government spending in improving human development outcomes by funding essential services such as healthcare, education, and infrastructure^9^. This result highlights the importance of effective public expenditure in achieving sustainable development goals. The coefficient for inflation is − 0.0248, which is statistically significant at the 1% level (*p* < 0.01). This negative relationship suggests that higher inflation rates are associated with lower iHDI. This result aligns with the view that high inflation can erode purchasing power and savings, adversely affecting economic stability and human development^10^. It underscores the importance of maintaining low and stable inflation to support human development. The coefficient for official development assistance (ODA) is 0.136, which is statistically significant at the 5% level (*p* < 0.05). This positive relationship suggests that higher levels of ODA are associated with higher iHDI, reflecting the potential benefits of foreign aid in supporting development projects and improving living standards^12^. This result indicates that, when effectively utilized, ODA can play a significant role in enhancing human development. The coefficient for FDI is 0.0518, which is statistically significant at the 1% level (*p* < 0.01). This positive relationship highlights the beneficial impact of foreign investment on human development. FDI can bring in capital, technology, and expertise that contribute to economic growth and improvements in living standards^13^. This result emphasizes the importance of attracting and effectively utilizing foreign investment to enhance human development. The coefficient for the first institutional quality composite index is 0.000209, but it is not statistically significant (*p* > 0.05). This suggests that, within this model, the first measure of institutional quality does not have a significant direct impact on HDI. However, institutional quality remains important for long-term development outcomes^11^. The coefficient for the second institutional quality composite index is 0.000273, but it is not statistically significant (*p* > 0.05). Similar to lncomp1, this result suggests that this measure of institutional quality does not have a significant direct impact on HDI within this model. The results from Model 7 highlight several important insights for policy formulation. The negative impact of national savings on HDI suggests that these savings need to be effectively managed and invested to avoid the resource curse. The positive impact of government expenditure on HDI underscores the importance of government spending on social services for sustained improvements in human development. Additionally, the beneficial impact of FDI on iHDI emphasizes the need for policies that attract and efficiently utilize foreign investment. The negative impact of inflation on iHDI indicates the importance of maintaining low and stable inflation to support human development. These insights align with the broader literature on economic development in Sub-Saharan Africa, emphasizing the complex interplay of savings, investment, and institutional factors in shaping human development outcomes^1–3^.

**Table 6:** Sequential GMM estimation results of National Savings and iHDI. *Source*: Author.

	(1) lnihdii	(2) lnihdii	(3) lnihdii	(4) lnihdii	(5) lnihdii	(6) lnihdii	(7) lnihdii
L. lnihdii	1.114 (·)	0.313 (·)	0.219 (·)	− 0.579 (·)	− 1.570 (·)	− 1.528*** (0.267)	− 1.243*** (0.342)
lnnatsavi	0.00299*** (1.49e−10)	0.0130 (·)	0.0103*** (1.16e−10)	0.00856*** (5.88e−11)	− 0.0918*** (4.78e−10)	− 0.0917*** (0.0199)	− 0.0660*** (0.0144)
lngovexi		0.382 (·)	0.445 (·)	0.803 (·)	1.093 (·)	1.070*** (0.0875)	0.986*** (0.136)
lninfi			− 0.00367*** 57e−11)	− 0.0128 (·)	− 0.0303 (·)	− 0.0297*** (0.00401)	− 0.0248*** (0.00427)
lnodai				0.0920 (·)	0.155 (·)	0.152*** (0.0172)	0.136*** (0.0260)
lnfdii					0.0730*** (2.48e−10)	0.0735*** (0.0158)	0.0518*** (0.0114)
lncompl						0.000411* (0.000165)	0.000209 (0.000141)
lncomp2							0.000273 (0.000230)
_cons	0.101 (·)	− 1.765 (·)	− 2.009 (·)	− 3.929 (·)	− 5.628 (·)	− 5.522*** (0.500)	− 5.025*** (0.732)
chi2	0	0	0	0	3.68979e+16	6099460.9	2124.0

Standard errors in parentheses *p < 0.05, ***p < 0.001.

Table 7 below shows the results of the analysis for MPI. In Model 1, the analysis of the determinants of the Multidimensional Poverty Index (MPI) begins with the coefficient for the lagged MPI (L.lnmpii), which is 0.353, though it is not statistically significant. This suggests that past values of MPI do not significantly predict current MPI, indicating that other factors might be influencing changes in MPI over time. This finding aligns with the literature that suggests the complexity of poverty dynamics, where historical poverty levels may not always directly influence current conditions due to various intervening factors^35^. The coefficient for national savings (lnnatsavi) is 0.251 but is not statistically significant, suggesting a limited direct impact of national savings on MPI in this model. This result points to the possibility that savings need to be effectively channeled into productive investments to impact human development meaningfully^1^. Government expenditure (lngovexi) has a coefficient of 0.255, statistically significant at the 1% level (*p* < 0.001). This positive relationship highlights the crucial role of government spending in improving MPI by funding social services and infrastructure, which are essential for enhancing living standards and reducing poverty^9^. In Model 2, the coefficient for lagged MPI (L.lnmpii) is 0.253, statistically significant at the 1% level (*p* < 0.001). This suggests that past MPI values positively influence current MPI, indicating some persistence in MPI levels. This persistence could be due to the entrenched nature of poverty, where past deprivation leads to continued hardship^35^. The coefficient for national savings (lnnatsavi) is 0.287, statistically significant at the 1% level (*p* < 0.001), indicating a positive impact of national savings on MPI. This suggests that higher savings can contribute to improving MPI when effectively utilized, supporting investments in critical areas such as education, healthcare, and infrastructure^1^. Government expenditure (lngovexi) also shows a positive coefficient of 0.255, statistically significant at the 1% level (*p* < 0.001), reaffirming its vital role in enhancing human development outcomes through adequate funding of essential services^9^. Model 3 presents a coefficient for lagged MPI (L.lnmpii) of 0.282, which is not statistically significant, suggesting limited predictive power of past MPI on current levels. This indicates that while there may be some persistence, it is not strong enough to be statistically confirmed in this model^35^. National savings (lnnatsavi) has a coefficient of 0.267 but is not statistically significant, suggesting that while savings are important, their direct impact on MPI may require effective investment strategies to be significant^1^. Government expenditure (lngovexi) has a coefficient of 0.349, which is also not statistically significant, suggesting that the impact of public spending on MPI may vary depending on how effectively the funds are utilized^9^. In Model 4, the coefficient for lagged MPI (L.lnmpii) is − 2.905, statistically significant at the 1% level (*p* < 0.001). This negative relationship suggests that high past MPI values are associated with significant improvements in current MPI, possibly due to targeted poverty reduction efforts in areas with historically high poverty^35^. National savings (lnnatsavi) has a positive coefficient of 0.207, statistically significant at the 1% level (*p* < 0.001), indicating that savings positively impact MPI. This underscores the importance of effective management and productive investment of savings to enhance human development^1^. Government expenditure (lngovexi) shows a negative coefficient of − 1.163, statistically significant at the 1% level (*p* < 0.001), suggesting that higher government spending may have adverse effects if not effectively managed. This could be due to inefficiencies or misallocation of funds^9^. Official Development Assistance (ODA) (lnodai) shows a strong positive coefficient of 3.220, statistically significant at the 1% level (*p* < 0.001), indicating that well-utilized aid can significantly enhance MPI by providing crucial support for development projects and essential services^12^. Model 5 shows similar patterns to Model 4, with the coefficient for lagged MPI (L.lnmpii) at − 2.901, statistically significant at the 1% level (*p* < 0.001), reinforcing the idea of diminishing returns or significant improvements in high-poverty areas^35^. National savings (lnnatsavi) has a coefficient of 0.199 but is not statistically significant, suggesting a positive but less certain impact on MPI^1^. Government expenditure (lngovexi) has a coefficient of − 1.175, statistically significant at the 1% level (*p* < 0.001), indicating potential inefficiencies in public spending^9^. The strong positive impact of ODA (lnodai) with a coefficient of 3.215, statistically significant at the 1% level (*p* < 0.001), highlights the critical role of foreign aid in enhancing human development when effectively managed^12^. In Model 6, the coefficient for lagged MPI (L.lnmpii) is − 3.279, statistically significant at the 1% level (*p* < 0.001), indicating strong diminishing returns in MPI. This result suggests that areas with high historical poverty levels might see substantial improvements due to targeted interventions^35^. National savings (lnnatsavi) has a coefficient of 0.141, statistically significant at the 1% level (*p* < 0.01), suggesting a positive impact on MPI^1^. Government expenditure (lngovexi) shows a negative coefficient of − 1.379, statistically significant at the 5% level (*p* < 0.05), indicating inefficiencies in public spending^9^. Inflation (lninfi) has a coefficient of − 0.0753, statistically significant at the 1% level (*p* < 0.01), indicating a negative impact of inflation on MPI. This highlights the adverse effects of high inflation on purchasing power and living standards^10^. ODA (lnodai) shows a strong positive coefficient of 3.610, statistically significant at the 1% level (*p* < 0.001), reaffirming the beneficial impact of well-utilized aid^12^. The coefficient for the first institutional quality composite index (lncomp1) is 0.0267 but not statistically significant, suggesting limited direct impact^11^. with all the variables in In Model 7, the analysis reveals several critical insights into the determinants of the Multidimensional Poverty Index (MPI) using the Generalized Method of Moments (GMM) sequentially to ensure robustness against endogeneity and other econometric issues. The coefficient for the lagged MPI (L.lnmpii) is − 3.163, which is statistically significant at the 1% level (*p* < 0.001). This significant negative relationship suggests that past MPI levels have a strong negative predictive power for current MPI levels. This result can be interpreted in the context of diminishing returns, where areas with previously high MPI might experience greater efforts and resources directed towards poverty reduction, leading to substantial improvements over time^35^. The coefficient for national savings (lnnatsavi) is 0.160, but it is not statistically significant (*p* = 0.115). Although not significant, the positive sign of the coefficient indicates a potential positive impact of national savings on MPI, aligning with the hypothesis that higher savings, when effectively utilized, can lead to improvements in human development outcomes by providing the necessary capital for investments in key sectors^1^. However, the lack of significance suggests that the direct impact of national savings might be overshadowed by other more immediate determinants of MPI in this model. Government expenditure (lngovexi) has a coefficient of − 1.321, statistically significant at the 5% level (*p* < 0.05). This significant negative relationship implies that higher government expenditure is associated with lower MPI, suggesting inefficiencies in public spending. This could indicate that despite high levels of expenditure, the allocation might not be optimal, or resources could be diverted due to corruption or mismanagement, failing to reach the intended beneficiaries and thus not translating into significant improvements in poverty reduction^9^. This finding underscores the importance of not just increasing public expenditure but also ensuring its effective and transparent utilization. The coefficient for inflation (lninfi) is − 0.0640, statistically significant at the 1% level (*p* < 0.01). This negative relationship suggests that higher inflation rates are associated with higher MPI, indicating that inflation adversely affects human development. High inflation can erode purchasing power and savings, destabilize economic conditions, and disproportionately impact the poor by increasing the cost of living and reducing real incomes, thereby exacerbating poverty levels^10^. This result highlights the critical need for maintaining low and stable inflation to support sustainable poverty reduction efforts. Official Development Assistance (ODA) (lnodai) shows a coefficient of 3.535, statistically significant at the 1% level (*p* < 0.001). This strong positive relationship suggests that higher levels of ODA are significantly associated with improvements in MPI, reflecting the potential benefits of foreign aid when effectively utilized. ODA can provide crucial financial support for development projects, improve access to essential services, and enhance infrastructure, thereby contributing to substantial reductions in poverty^12^. This finding indicates that foreign aid, when managed effectively and directed towards critical areas, can play a pivotal role in enhancing human development outcomes. The Institutional Quality Composite Index 1 (lncomp1) has a coefficient of − 0.00546, which is not statistically significant (*p* = 0.0767). Although the coefficient is not significant, the negative sign suggests a potential adverse impact of institutional quality on MPI in the short term. This could reflect the transitional costs associated with institutional reforms, where the benefits of improved governance structures might take time to materialize^11^. Effective institutional reforms are essential for long-term development, but they need to be carefully managed to mitigate any short-term negative impacts. The Institutional Quality Composite Index 2 (lncomp2) has a coefficient of − 0.00837, statistically significant at the 5% level (*p* < 0.05). This significant negative relationship suggests that higher institutional quality, as measured by this index, is associated with lower MPI, indicating that better institutional quality can contribute to poverty reduction. Strong institutions can enhance the effectiveness of public policies, reduce corruption, and improve the delivery of public services, thereby positively impacting human development^11^. Overall, the findings from Model 7 underscore the complex interplay between various determinants of human development. The significant negative impact of lagged MPI highlights the importance of historical context in understanding current poverty levels. The mixed results for national savings and government expenditure suggest that while financial resources are crucial, their effective and efficient utilization is paramount. The adverse effects of inflation underscore the need for stable macroeconomic conditions to support poverty reduction. The positive impact of ODA reaffirms the potential of foreign aid in enhancing development outcomes when effectively managed. Finally, the results for institutional quality indices highlight the critical role of good governance and strong institutions in sustainable human development.

**Table 7:** Sequential GMM estimation results of National Savings and MPI. *Source*: Author.

	(1) lnmpii	(2) lnmpii	(3) lnmpii	(4) lnmpii	(5) lnmpii	(6) lnmpii	(7) lnmpii
L. lnmpii	0.353 (·)	0.253*** (1.73e−09)	0.282 (·)	− 2.905*** (5.10e−09)	− 2.901*** (1.54e−08)	− 3.279*** (0.258)	− 3.163*** (0.750)
lnnatsavi	0.251 (·)	0.287*** (1.50e−09)	0.267 (·)	0.207*** (7.78e−10)	0.199 (·)	0.141*** (0.0381)	0.160 (0.115)
lngovexi		0.255*** (2.73e−10)	0.349 (·)	− 1.163*** (5.82e−09)	− 1.175*** (4.69e−09)	− 1.379*** (0.176)	− 1.321* (0.528)
lninfi			− 0.0195 (·)	− 0.0620 (·)	− 0.0626 (·)	− 0.0753*** (0.00530)	− 0.0640*** (0.0171)
lnodai				3.220*** (8.81e−09)	3.215*** (1.37e−08)	3.610*** (0.259)	3.535*** (0.708)
lnfdii					0.00601 (·)	0.0267 (0.0190)	− 0.000546 (0.0767)
lncompl						− 0.00885 (0.00453)	− 0.00837** (0.00288)
lncomp2							− 0.00283 (0.00230)
_cons	− 1.441 (·)	− 2.309*** (6.32e−09)	− 2.414 (·)	− 7.735 (·)	− 7.673*** (3.13e−09)	− 8.131*** (0.237)	− 8.056*** (0.562)
chi2	0	3.78442e+17	0	7.43616e+17	3.56477e+16	25709.7	1661.9

Standard errors in parentheses *p < 0.05, **p < 0.01, ***p < 0.001.

Overall, the hypothesis testing revealed mixed results regarding the association of national savings with improvements in human development indices. Hypothesis H1 posited that higher national savings rates would be associated with improvements in Human Development Index (HDI) scores. However, the findings indicate that while national savings had some positive impacts on HDI in certain models, the results were not consistently statistically significant across all models. Hypothesis H2, which proposed that higher national savings rates would improve Inequality-adjusted HDI (iHDI) scores, also yielded mixed results. The positive impact of national savings was observed in some instances but lacked consistent statistical significance, indicating that the relationship between national savings and iHDI is complex and depend on how savings are utilized and managed within the broader economic framework. Hypothesis H3, suggesting that higher national savings rates would be associated with a reduction in the Multidimensional Poverty Index (MPI), found some support in the data. National savings exhibited a positive impact on reducing MPI, particularly when effectively channeled into developmental projects and investments that directly benefit the poor. However, the relationship was not universally strong across all models, underscoring the need for comprehensive strategies that encompass effective utilization of savings alongside other developmental initiatives.

## Conclusion

The findings of this study provide a nuanced understanding of the impact of national savings on economic development, specifically focusing on the ten poorest countries in Sub-Saharan Africa; employing HDI, iHDI, and MPI as principal indicators. The findings reveal a significant impact (but inconsistent across the models) on national savings rates by improvements in HDI and iHDI scores, underscoring the positive influence that enhanced savings practices can have on socio-economic development. The analysis reveals that national savings, contrary to the initial hypotheses, do not have a straightforward positive impact on human development indices (HDI, iHDI, and MPI). However, the relationship between national savings and multidimensional poverty, as measured by MPI, emerges as more intricate, shedding light on the complex interplay between economic variables and poverty levels. overall, the results suggest that national savings may not be effectively utilized for productive or welfare-enhancing activities in these countries. This aligns with the theoretical perspectives of Harrod^6^ and Domar^5^, who emphasized the importance of effective investment of savings for economic growth. The study also highlights the significant positive impact of government expenditure and foreign direct investment (FDI) on human development, underscoring the importance of strategic investments in public goods and foreign capital. Conversely, the mixed effects of inflation and official development assistance (ODA) emphasize the need for stable economic policies and effective utilization of foreign aid. The modest positive impact of institutional quality suggests that improvements in governance and institutional frameworks can contribute to human development, although further investigation is needed to understand the specific components of institutional quality that impact HDI, IHDI and MPI.

## Policy recommendations

Based on the study's findings, several policy recommendations can be made to enhance the impact of national savings on economic development in Sub-Saharan Africa. Firstly, governments should focus on creating an enabling environment for official savings and the effective utilization of national savings. This includes implementing policies that promote financial inclusion, improve access to credit, and encourage savings among the population and the governments as well. Additionally, there should be a concerted effort to channel national savings into productive investments that can drive economic growth and development. Secondly, strategic investments in public goods and services are crucial. Governments should prioritize spending on education, healthcare, infrastructure, and social protection programs. These investments can have a significant positive impact on human development and help to reduce poverty and inequality. It is also important to ensure that public expenditure is efficient and transparent, with mechanisms in place to prevent corruption and mismanagement of resources. Thirdly, attracting foreign direct investment (FDI) should be a key policy objective. FDI can bring in much-needed capital, technology, and expertise, which can contribute to economic development and job creation. Governments should create a conducive environment for FDI by improving the ease of doing business, providing incentives for foreign investors, and ensuring political and economic stability. Fourthly, maintaining macroeconomic stability is essential for sustainable development. High inflation can erode purchasing power and negatively impact living standards. Therefore, central banks should adopt prudent monetary policies to control inflation and ensure macroeconomic stability. This includes maintaining a stable exchange rate, managing public debt effectively, and implementing sound fiscal policies. Fifthly, effective management of official development assistance (ODA) is crucial. Governments should ensure that ODA is effectively managed and directed towards critical areas that can drive human development. This includes investing in infrastructure, education, healthcare, and poverty reduction programs. Reducing dependency on foreign aid and promoting self-sufficiency should also be key policy objectives. Lastly, strengthening institutional quality is essential for effective governance and the efficient utilization of resources. Governments should focus on improving the rule of law, reducing corruption, and enhancing the capacity of public institutions. Better institutional quality can contribute to poverty reduction and improved human development outcomes. By implementing these policy recommendations, Sub-Saharan African countries can enhance the impact of national savings on economic development and achieve sustainable improvements in human development indices.

## Data Availability

The database supporting the results of this research work will be made available on reasonable requests from the corresponding author.
